# Indoor-Breeding of *Aedes albopictus* in Northern Peninsular Malaysia and Its Potential Epidemiological Implications

**DOI:** 10.1371/journal.pone.0011790

**Published:** 2010-07-27

**Authors:** Hamady Dieng, Rahman G. M. Saifur, Ahmad Abu Hassan, M. R. Che Salmah, Michael Boots, Tomomitsu Satho, Zairi Jaal, Sazaly AbuBakar

**Affiliations:** 1 School of Biological Sciences, Universiti Sains Malaysia, Penang, Malaysia; 2 Department of Animal and Plant Sciences, University of Sheffield, Sheffield, United Kingdom; 3 Faculty of Pharmaceutical Sciences, Fukuoka University, Fukuoka, Japan; 4 Department of Medical Microbiology, University of Malaya, Kuala Lumpur, Malaysia; State University of Campinas (UNICAMP), Brazil

## Abstract

**Background:**

The mosquito *Ae. albopictus* is usually adapted to the peri-domestic environment and typically breeds outdoors. However, we observed its larvae in most containers within homes in northern peninsular Malaysia. To anticipate the epidemiological implications of this indoor-breeding, we assessed some fitness traits affecting vectorial capacity during colonization process. Specifically, we examined whether *Ae. albopictus* exhibits increased survival, gonotrophic activity and fecundity due to the potential increase in blood feeding opportunities.

**Methodology/Principal Findings:**

In a series of experiments involving outdoors and indoors breeding populations, we found that *Ae. albopictus* lives longer in the indoor environment. We also observed increased nighttime biting activity and lifetime fecundity in indoor/domestic adapted females, although they were similar to recently colonized females in body size.

**Conclusion/Significance:**

Taken together these data suggest that accommodation of *Ae. albopictus* to indoor/domestic environment may increase its lifespan, blood feeding success, nuisance and thus vectorial capacity (both in terms of increased vector-host contacts and vector population density). These changes in the breeding behavior of *Ae. albopictus*, a potential vector of several human pathogens including dengue viruses, require special attention.

## Introduction

The acquisition of indoor-breeding behavior can potentially increase the biting activity of mosquito vectors that opportunistically bite humans outdoors during the day. This may therefore have important implications to disease transmission. However, despite this epidemiological importance, there have been no previous studies of this issue in dengue vectors.

Dengue viruses infect up to 50 million people each year, causing more than 20,000 deaths [1], [2]. These flaviviruses are mainly transmitted by *Aedes aegypti*, but also *Ae. albopictus* [3]. Native to the Oriental Region and some islands in the Indian Ocean [3], *Ae. albopictus* has become well-established in the Western hemisphere where it is the second main vector of dengue [4]. It is also an important vector of yellow fever and various types of encephalitis virus, as well as a competent vector of at least 23 other arboviruses under laboratory conditions [5], [6], [7]. It is well adapted to peridomestic environments with its larvae breeding in artificial containers and adults aggressively biting humans and different animals during the day [3].

Efforts to control dengue have mainly involved insecticide spraying programs, but this strategy has proven ineffectual [8]. While a vaccine is currently under development, without immediate prospects for success, vector control remains the only viable method to prevent dengue transmission [9], [10], [11], [12]. Improved knowledge regarding egg-laying behavior is relevant because it underpins the primary surveillance method, i.e., ovitraping [13], [14]. However, the most commonly used ovitrap, the CDC gravid trap, is not appropriate for capturing *Ae. albopictus* [15], [16].

Blood feeding in mosquitoes represents phenotypic expression of reproductive investment as it is the acquisition of resources specifically for reproduction [17]. Reproductive output represents the energy allocated to egg production and oviposition that could otherwise be allocated to maintenance of somatic function, and the act of oviposition is associated with a risk to survival [18]. There has been a great deal of research regarding the variations of reproductive investment and outcome. Overall, increases in both number and size of blood meals result in increased individual egg mass and number of eggs [18]. Clearly, in the field an increased frequency of blood uptake will tend to require host – mosquito contact and expose hosts to a greater risk of disease transmission.

*Ae. albopictus* has been occasionally incriminated in dengue epidemics in Asian countries [19], [20], [21]. The first report of a dengue epidemic in Malaysia was on Penang Island, and dengue fever with hemorrhagic manifestations was also reported from this Island [22]. These epidemics were accompanied by increased populations of *Ae. albopictus*. Increased invasiveness [23] and increased population densities of [24] have been recently been reported in the Malaysian peninsula. *Ae. albopictus* females was commonly observed developing in indoor/domestic sites throughout Penang Island during our 2009 entomological survey at Balik Pulau (Sungai Pinang and Sg. Burung), Gelugor, Jelutong, Air Itam (Kampung Relau), and other sites. This shift from outside to inside human dwellings is likely to increase the opportunities for females to obtain blood meals. This indoor immature breeding has raised the question of whether this mosquito may exhibit increased gonotrophic activity (GA) in response to potentially greater blood meal sources.

Given the potentially crucial interactions between reproduction, blood feeding, and vectorial capacity, we examined the GA and fecundity of *Ae. albopictus* using females derived from wild mosquitoes collected from outdoor containers in Kampung Teluk Tempoyak and Balik Pulau, Malaysia, with their daughters after they had spent five generations under laboratory conditions.

## Materials and Methods

### Occurrence of *Ae. albopictus* larvae in indoor containers

**Figure 1:**
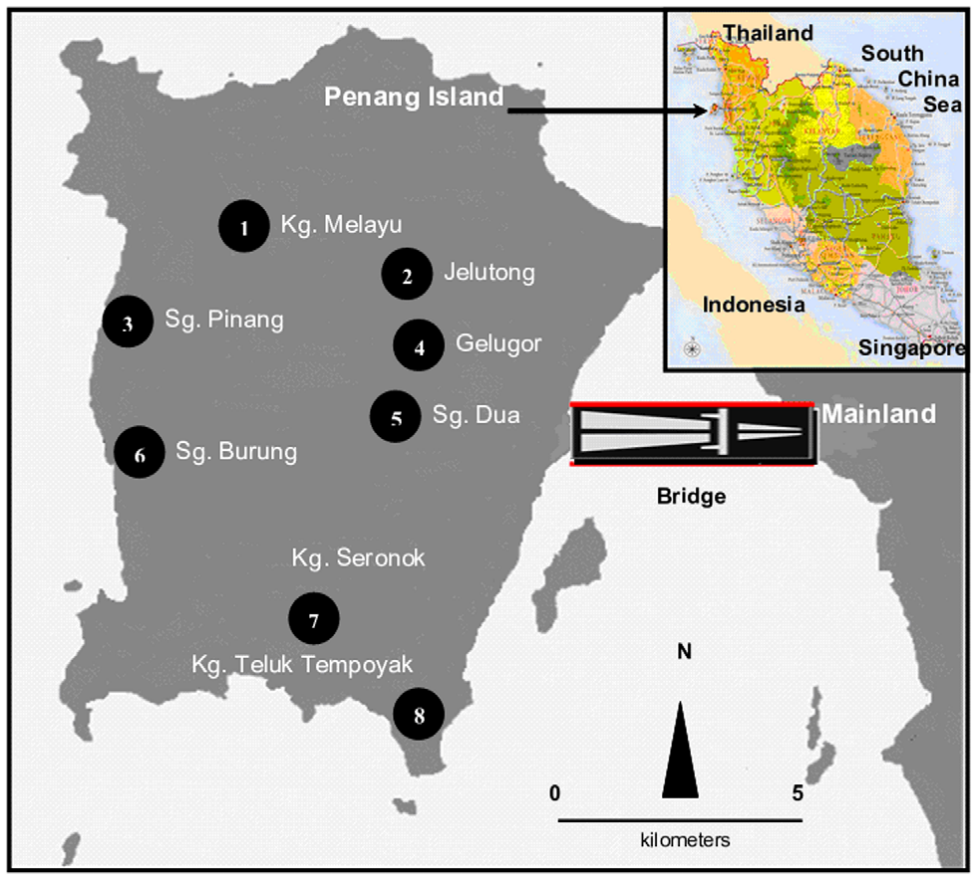
Map of Malaysia and locations of the *Aedes* survey areas on the Island of Penang.

A survey of *Aedes* was carried out from February to June 2009 in Penang province, Malaysia, located between latitudes 5°8′N and 5°35′N and longitudes 100°8′E and 100°32′E [25], covering nine residential areas (townships and villages) on Penang Island (Figure 1). The survey zone is surrounded by hills on the northwest with the rest of the zone opening up into low-lying areas occupied by human populations. Immature mosquito stages were collected in household containers.

### Statement on ethic issues

This study was carried out in accordance with the principles expressed in the Declaration of Helsinki. The study was approved by the Biological Research Ethics Committee at University Sains Malaysia (Projects # 07-05-16-MG1-GMB15, # 1001/PBIOLOGI/842004 and Fellowship grant # RU:1001/229/29301/CIPS/AUPE001).

### Colonization of wild *Ae. albopictus*

The *Aedes* mosquitoes used in this study were derived from wild pupae collected from outdoor containers in the survey zone. A colony was established in the insectarium at the School of Biological Sciences, Universiti Sains Malaysia, Penang. Larvae were reared routinely on a diet of dried yeast. Adults had access to 10% glucose solution, and females were blood-fed at two days old on restrained mice. Eggs were processed, dried, and stored under laboratory conditions (temperature 29°C±3°C, relative humidity 75%±10% and photoperiod 13:10 h, 1h dusk provided by an electrical lighting from a 60 and 25-watt incandescent bulbs.

### Bioassays

Bioassays were carried out using females derived from wild mosquitoes (FWMs) and females derived from FWMs after five generations (*d5*FWMs) under laboratory conditions. In all bioassays, disposable plastic cups (9×11.5 cm) filled with 30 mL of tap water and lined with filter papers (#1, Whatman®; Whatman International, Maidstone, UK) as an oviposition substrate, were used to provide females with sites for egg deposition. The filter papers were folded into a double-chambered cone and placed such that the mosquitoes could lay their eggs inside or outside the cone. A sugar meal source was supplied from cotton wads soaked in 10% glucose solution placed at the top of the cages. A rearing cage containing 100 males was used as a mating source for experimental females. Male cage was supplied with individual males from field-collected pupae.

### Experiments

The first experiment was performed to determine the oviposition responses of wild *Ae. albopictus* in the laboratory in relation to blood feeding time. Fifty two-day-old FWMs were blood-fed at 09:00 am on restrained mice. Similarly, a group of fifty two-days old FWMs were offered blood meals on the same day, but at 17:00. In both cases, females were provided with 10% glucose solution for two days. After they became gravid, they were placed individually in oviposition cages holding oviposition cups. Egg deposition was checked two days later at five time points during the day starting at 08:00 (09:00, 11:30, 17:00, 20:30, and 23:30). At each time point, cups with eggs were removed, eggs were counted, and new cups were placed in the cages.

The second experiment was performed to examine the nocturnal biting activity of wild *Ae. albopictus*. Ten newly emerged FWMs were maintained on 10% glucose solution for 3 to 4 days, and thereafter starved for a brief period (12 h). The mosquitoes were then placed in cages containing a restrained mouse and observed continuously. Ten females of *Ae. aegypti* were treated as described above and used as a control group regarding its endophagic behavior [26] and nighttime biting activity [27], [28]. Engorgement was checked at eight time points during the night (20:00, 21:00, 22:00, 23:00, 24:00, 01:00, 02:00 and 03:00), and the feeding times were recorded for each female of both species. The experiment was run under laboratory photoperiod conditions.

The third experiment involved determining the effects of *Ae. albopictus* domestication time on the number of gonotrophic cycles (GCs) it can perform in its lifetime. A total of one hundred fully blood-fed FWMs and *d5*FWMs were used in this experiment. Each gravid female was placed singly in an oviposition cup holding a 10% glucose solution source. Egg deposition was confirmed by examining filter papers under a dissecting microscope at the end of the oviposition period. The same females were again given access to blood re-feeding, and provided with oviposition cups, which was repeated until their death. In addition to the possibility that the generation rank affects the number of times eggs are produced, we also examined whether there was any effect of generation rank on lifetime fecundity. Eggs oviposited in each GC of both FWMs and *d5*FWMs were counted by examining filter papers under a dissecting microscope. Body size was measured in all females used in the fecundity experiment due to the possibility that this parameter affects the number of eggs produced.

### Data collection and analysis

Immature *Aedes* (larvae and pupae) collected from indoor containers were identified morphologically with reference to appropriate taxonomic keys [29]. In all bioassays, females in the experimental group were allowed to feed on blood for one day, to digest it for two days, and to lay eggs for 24 h. Blood feeding was scored based on distention of the abdomen. We recorded the number of GCs for each experimental female (FWMs and *d5*FWMs). Following previous studies [30], we considered a GC as the time between ingestion of blood and commencement of oviposition. In each GC, the number of eggs deposited was used as the score of fecundity, thus adopting others [31]. Eggs were counted under a dissecting microscope (Meiji EMZ; Meiji Techno Co. Ltd, Tokyo, Japan). We considered percentage female survival as the number of individuals that laid eggs divided by the initial number of individuals tested in each GC trial. As an indicator of body size, the length of one wing per female from the axillary incision to the apical margin excluding the fringe scales was measured under a light microscope (Olympus CX41; Olympus, Tokyo, Japan) as described by others [32]. The rate of oviposition was determined as the number of females that oviposited divided by the initial number of females tested. The differences in the number of GCs, fecundity, and body size between FWMs and *d5*FWMs were examined by analysis of variance using the statistical software package Systat v.11 (Systat Software, Inc., Richmond, CA, USA) [33]. In the fecundity experiment, Tukey's test was applied to separated means. In all statistical analyses, *P*<0.05 was taken to indicate statistical significance. Survival rates were compared based on differences in percentages.

## Results

### Survey *Aedes* inside houses

**Figure 2:**
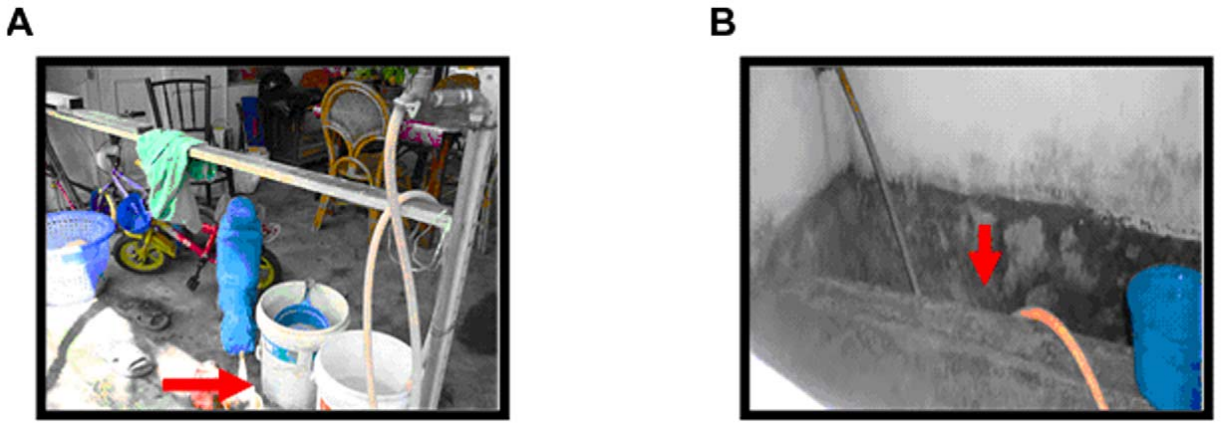
Indoor breeding of *Ae. albopictus*. Empty paint containers (A) and a cement tank (B) in residences in the township of Gelugor, Penang, Malaysia contained high numbers of larvae. Red arrows indicate the containers and tank.

Indoor containers with immature *Aedes* stages consisted of drums, flower vases, ant-guards, buckets, cement tanks, empty paint cans, wells, hare pots, underground floor, and sinks (Table 1, Figure 2). The majority of mosquitoes collected were *Ae. albopictus*, but *Ae. aegypti* was also found. Between February and May, the sizes of immature stage populations remained high and constant in the five residential areas of the study. In some cases, larval instars of *Ae. albopictus* were persistent until June, suggesting that oviposition events did occur continuously (Table 1). The heterogeneity of larval developmental stages and their persistence suggested that *Ae. albopictus* has established populations within peoples' houses.

**Table 1:** Characteristics of *Ae. albopictus* collections from indoor/domestic containers from homes throughout Penang Island in 2009.

Date	Location	Household containers types	First	Second	Third	Fourth	Pupae	Species
24/02/09	Sg. Burung, BP	Drums, Buckets	300	350	275	200	130	*Ae. albopictus*
24/03/09	Kg. TT	Drums, Cement tanks, Empty paint cans	63	100	190	268	55	*Ae. albopictus*
31/03/09	Sg. Pinang	Drums, Cement tanks	68	137	49	40	26	*Ae. albopictus*
28/04/09	JLN Baru, BP	Ant-guards	60	220	120	200	50	*Ae. albopictus*
5/5/2009	Kg. Melayu, AI	Drums, Buckets	130	195	75	110	40	*Ae. albopictus*
19/05/09	Kg. Seronok	Floor vases	0	5	10	8	2	*Ae. albopictus*
19/05/09	Sg. Dua	Wells	100	180	70	80	3	*Ae. aegypti*
26/05/09	JLN Baru, BP	Empty paint cans	0	10	15	45	0	*Ae. albopictus*
23/06/09	Kg. Seronok	Buckets	0	0	10	6	0	*Ae. albopictus*
23/06/09	Jelutong	Hare pots	0	30	20	0	0	Mixed with *Ae. aegypti*
9/6/2009	Gelugor	Cement tanks	50	35	25	60	0	*Ae. aegypti*
16/06/09	JLN Baru, BP	Empty paint cans	20	25	10	0	0	*Ae. albopictus*
30/06/09	Jelutong	Underground floor	200	300	500	570	50	Mixed with *Ae. aegypti*
9/6/2009	Jelutong	Sinks	20	35	10	0	0	*Ae. aegypti*

### Oviposition activity of wild *Ae. albopictus*

**Figure 3:**
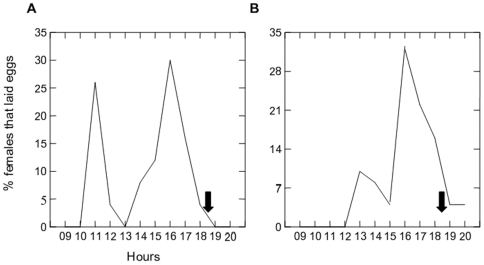
Oviposition responses of FWMs *Ae. albopictus* offered blood meals at 09:00 (A) and 17:00 (B) of the day. The arrow indicates the 1h dusk period.

Figure 3 shows the oviposition responses of FWMs of *Ae. albopictus* given blood meals at two different times of the day. In both cases, oviposition activity was low to absent during the night. *Ae. albopictus* exhibited two distinct peaks of oviposition activity for all feeding times: a narrow peak in the morning and a wider peak during the afternoon. Females that took a blood meal in the early morning (09:00, Figure 3A) showed a narrow peak of oviposition at 11:00 two days later. Those that fed on blood in the late afternoon (17:00, Figure 3B) showed a weak and narrow peak around noon (13:00) and a larger wider peak in the afternoon (from 15:00 to 18:00).

### Patterns of nocturnal blood feeding

The hourly nighttime biting activities of *Ae. albopictus* and *Ae. aegypti* were examined between 20:00 and 03.00. *Ae. albopictus* showed a constant pattern of biting activity at all time points examined, with higher percentage fed and longer feeding time in comparison to *Ae. aegypti* (Table 2).

**Table 2:** Nocturnal biting frequencies of FWMs of *Ae. albopictus* and *Ae. aegypti*.

	FWMs *Ae. albopictus*		FWMs *Ae. aegypti*	
	% fed	Time	% fed	Time
8pm	100	2′	90	5′
9pm	100	2′	90	10′
10pm	100	2′ 30	80	6′
11pm	100	2′	90	6′
12pm	100	3′	80	7′
01am	100	2′.30	80	9′
02am	100	3′	90	6′
03am	100	3′	70	8′

*Time (in minutes after which all 10 individuals have taken a blood meal.

### Survival and gonotrophic activity period

**Figure 4:**
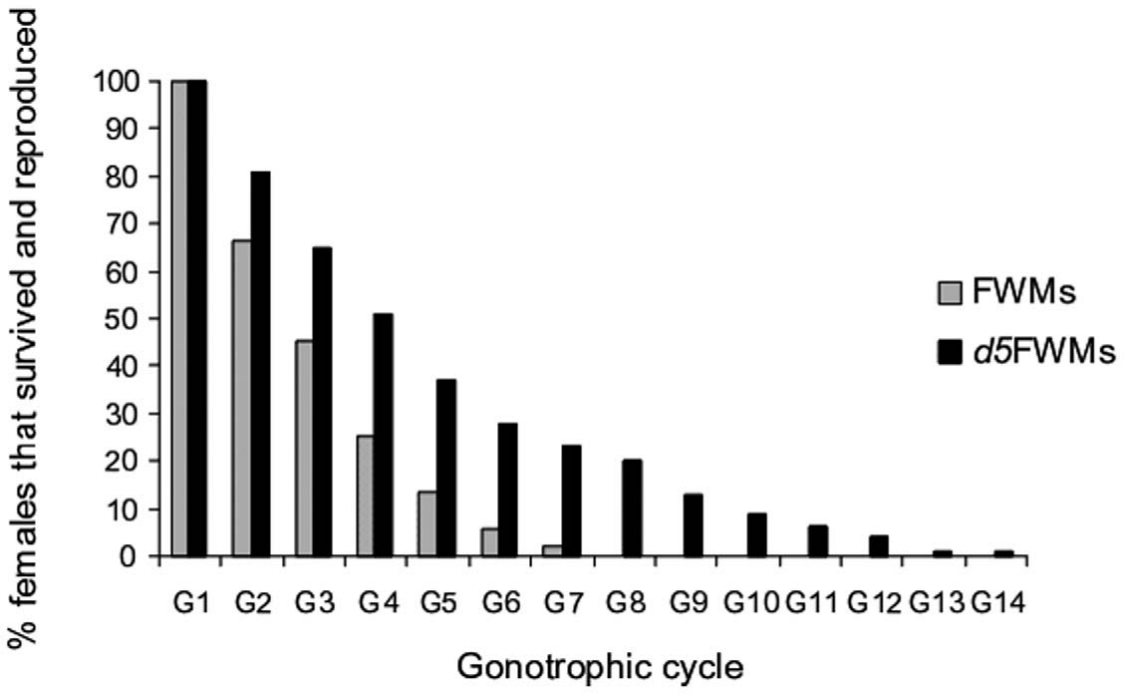
Percentages of surviving FWMs and *d5*FWMs of *Ae. albopictus* and their gonotrophic activity periods.

The survival rate decreased when progressing from first to last GC for both FWMs and *d5*FWMs, although the pattern of the decrease varied with female generation. In FWMs, more than 50% of the females examined died at the third GC. Among the only two FWMs that survived and achieved a seventh GC, none showed subsequent survival. In *d5*FWMs, more than 50% of females survived and achieved a fourth GC. More than 20% of *d5*FWMs survived and reproduced a seventh time. Among these, six individuals reproduced an eleventh time and one individual reproduced a fourteenth time. Overall, survival decreased with time, but this was more pronounced among FWMs (Figure 4).

### Patterns of gonotrophic activity

**Figure 5:**
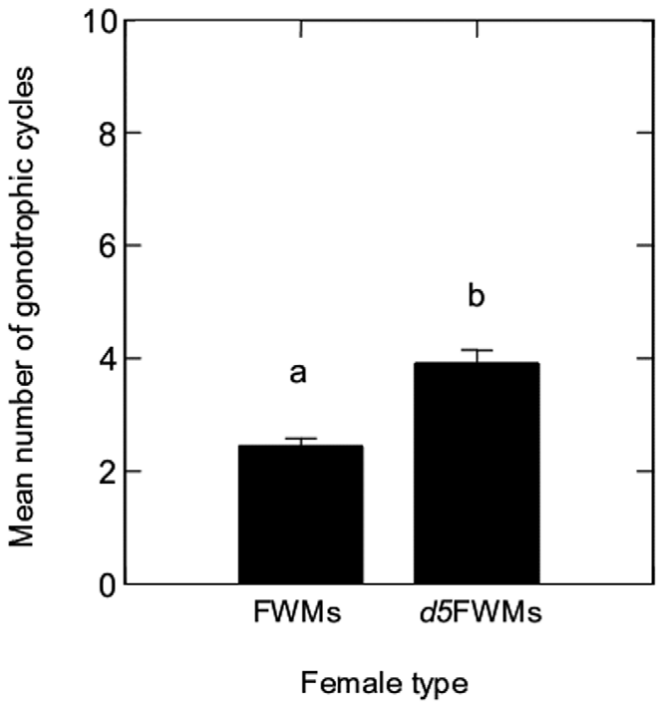
Numbers of gonotrophic cycles (mean ± SD) of FWMs and *d5*FWMs of *Ae. albopictus*. Bars labeled with the same letter are not significantly different (*P*<0.05).

The number of GCs achieved by *Ae. albopictus* varied significantly with generation (F = 30.06, df = 1, *P*<0.001). The mean number of cycles for FWMs was 2.0±0.10 (range: 1–7), while that in *d5*FWMs was 4.0±0.10 (range: 1–14). Therefore, it is clear that females from *d5*FWMs had a greater number of GCs (Figure 5).

### Fecundity

**Figure 6:**
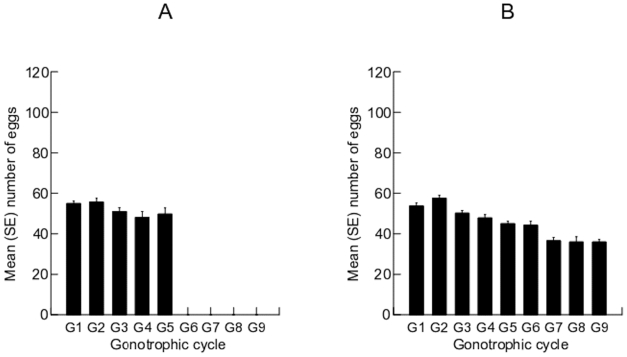
Numbers of eggs (mean ± SE) laid by FWMs (A) *d5*FWMs (B) of *Ae. albopictus*.

Figures 6A and 6B show the egg deposition patterns in FWMs and *d5*FWMs of *Ae. albopictus*, respectively. The overall egg production did not differ significantly between females from the two generations (Table 3). Pairwise comparisons using the Tukey test revealed no significant difference in egg production during the first (F = 0.015, df = 1, *P* = 0.905), second (F = 0.041, df = 1, *P* = 0.842), third (F = 0.195, df = 1, *P* = 0.663), fourth (F = 0.169, df = 1, *P* = 0.684), or fifth (F = 0.001, df = 1, *P* = 0.975) GC.

**Table 3:** Statistical analysis by ANOVA of the variations in fecundity and body size between FWMs and *d5*FWMs of *Ae. albopictus*.

	df	F-ratio	P
Fecundity			
FWMs	4	0,707	0.591
*d5*FWMs	8	2.39	0.014
Body size	1	1.049	0.316

In FWMs, the level of egg production was maximal in the first GC (63.25±8.00). The number of eggs laid was not significantly different between the different GCs achieved (F = 0.707, df = 4, *P* = 0.591). There were more eggs deposited by FWMs at the first than at the fifth GC, but the difference in mean egg deposition between the first and last GCs was not significant (F = 2.001, df = 1, *P* = 0.171).

In *d5*FWMs, the peak of egg production was recorded at the third GC (65.77±8.60), and egg production varied significantly between GCs in these mosquitoes (Table 1). From the third GC, egg production tended to decrease as the rank of GC progressed. The mean number of eggs deposited by *d5*FWMs was significantly lower at the ninth than at the first GC (F = 7.444, df = 1, *P* = 0.012).

### Body size

**Figure 7:**
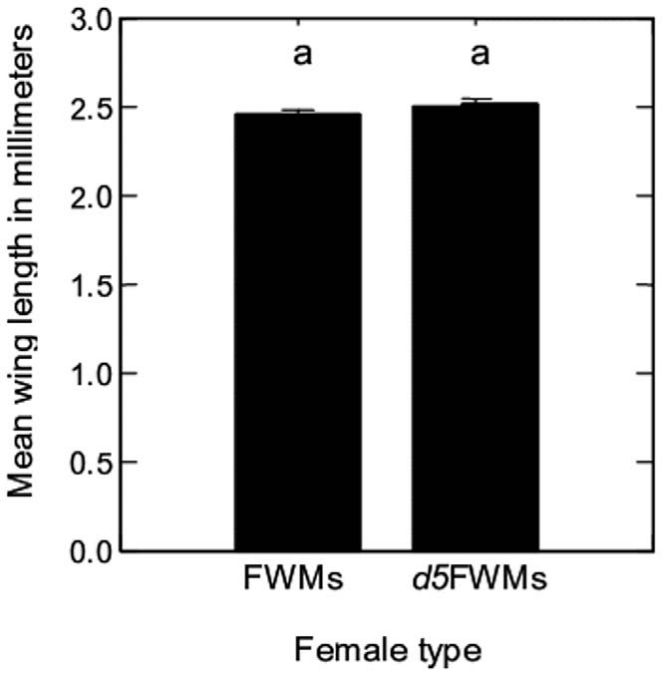
Wing length (mean ± SE) of FWMs and *d5*FWMs of *Ae. albopictus*. Bars labeled with the same letter are not significantly different (*P*<0.05).

The mean wing lengths were 2.46±0.04 and 2.51±0.04 mm in FWMs and *d5*FWMs, respectively. Although *d5*FWMs tended to be slightly larger than FWMs, there was no significant difference in mean wing length between the two groups (Table 1, Figure 7).

## Discussion

The most important observation in the present survey was that *Ae. albopictus* breeds inside peoples' houses in many parts of Penang Island, Malaysia. Larval populations were heterogeneous and most developmental stages were present over the five-month period of the survey. As this mosquito typically shows outdoor breeding behavior [34], [3], [35], the persistence of its larval and pupal stages in indoor containers over a long period suggests that *Ae. albopictus* is being adapted to the indoor environment. A similar observation was recently reported in neighboring Thailand [36], but there have been no studies regarding the epidemiological significance of these observations. Here, we examined the gonotrophic performance of wild *Ae. albopictus* with regard to the crucial interactions between biting activity and vectorial capacity [37].

As the present study was begun with a wild population, it was first necessary to determine whether they could oviposit under laboratory conditions. Oviposition trials indicated that FWMs can lay eggs in the laboratory and that the patterns of egg–laying were associated with blood feeding time. The findings of the present study were consistent with the natural oviposition behavior of this mosquito [38], [39], [40], [41], therefore allowing the long-term experiments required for this study.

We found a major effect of level of adaptation to indoor/domestic environment on the number of GCs in the laboratory. *d5*FWMs showed a much higher number of GCs than their FWMs counterparts. In mosquitoes, the nutritional history of the parents is influential in determining the fecundity of daughters [42]. This study showed that daughters from parents reared in a food-limited environment produced more eggs than those from parents reared under high food conditions. They suggested that this increased fecundity arose to compensate for expected decreased longevity in stressing environments. Here, females derived from wild mosquitoes (FWMs) and females derived from these FWMs after five generations achieved 7 and 14 GCs, respectively. *d5*FWMs survival rate was higher than that of FWMs mosquitoes. Although the mechanisms underlying these observations are not yet clear, they could be the result of at least two processes. First, the highly nutritious food conditions in the laboratory could lengthen the mosquito lifespan, and thus increase the probability that it reproduces. Second, the shift from a complex wild environment to a simple environment, such as that in the laboratory, may result in physiological changes that increase the allocation of energy to functions other than egg production, thereby increasing the probability of survival. Longer living females may take more blood meals and reproduce more simply because of the increased availability of meals. Epidemiologically, an increased number of GCs will tend to increase the probability of disease occurrence. With an extended period of GA, females have a higher probability of picking up and transmitting a disease agent as well as an increased lifespan as blood provides an alternative energy source for survival [43]. The increased period of reproduction of the *d5*FWMs will also lead to a higher mosquito population density, which is also likely to be associated with increased occurrence of disease. Population levels expressed as larval [44], [45], [46], pupal [47], and adult indices [48] are often associated with levels of risk for dengue transmission. Dengue outbreaks occurred in Singapore [49] but not in Brazil [50] when the national overall percentage of houses positive for larvae (HI) was below 1%. In Puerto Rico, the incidence of dengue increased one month after larval density peaked [51], whereas in Brazil, dengue seroincidence increased when the HI was above 3% [52]. The present study was prompted by the permanent presence of biting adults and immature stages of *Ae. albopictus* within residences in Teluk Tempoyok and Balik Pulau located in northern peninsular Malaysia and a lack of information regarding the epidemiological implications. Although we did not determine whether these populations were infected with dengue viruses, the focal point of this study was that the presence of larvae strongly suggests that at least a GC has been achieved. Thus, infection would occur if the virus was present. Note that this species is competent for many viruses [6], [7], but has only occasionally been incriminated in minor dengue epidemics all the world, e.g., in Hawai in 2001–2002 [53].

In FWMs and *d5*FWMs, the number of eggs oviposited tended to decrease as GC rank progressed, but this effect was most marked in the second group. As in most anautogenous mosquitoes, the production of eggs in *Ae. albopictus* requires the ingestion of blood [54], [55]. The female converts about 20% of the ingested blood meal into egg constituents [56]. Several groups have reported that the degree to which eggs are produced depends largely on meal size [32], [57], [58]. Indeed, a female that ingests a large blood meal size will tend to invest more in egg production than a female with a small blood meal. In the present study, we have used females adapted to laboratory conditions and females derived from wild pupae. It is often assumed that wild insects are subject to much harsher environmental conditions that trigger small body size and that they have lower levels of energetic reserves than laboratory strains. Clearly, in the laboratory, the highly nutritious larval diet will tend to produce large bodied mosquitoes capable of blood feeding for long periods due to little or no host defense behavior from anesthetized hosts [32]. Here, FWMs and *d5*FWMs were similar in body size and had the same feeding time, so differential egg production due to differences in meal size is unlikely. These discrepancies may be explained by differential utilization of blood. Adult mosquitoes feed on blood for immediate energy needs [57], [59], but in some cases, they use blood as an alternative energy source for survival [43]. There is evidence that colonization alters reproductive traits [60], in particular offspring fecundity [42]. Therefore, it is tempting to suggest that the accommodation of the wild strain to the laboratory environment has occurred in addition to physiological changes relative to blood use. It is possible that the reduced level of egg production observed with increasing generations represented a compensation for better acclimation to the laboratory environment. Presumably, protein use by *d5*FWMs offsets the costs associated with egg production and facilitates population maintenance in this environment. In support of this suggestion, it has been reported that *Ae. albopictus* may use some blood proteins for maintenance [61].

The pattern of egg production was similar between females of both types, but lifetime fecundity was greater in *d5*FWMs. This difference was the result of their greater survival. Females with a long lifespan may take more blood meals and reproduce more simply because of the increased availability of meals. Easy access to blood sources in any host – vector interaction can be of crucial epidemiological significance because increased frequency of host-biting may favor the spread of infectious disease present. The presence of *Ae. albopictus* inside houses, that was observed in many residences throughout Penang Island, appears to facilitate human blood feeding. In this context, biting activity during both the day and night may increase mosquito – human contact. In addition, dengue viruses can be transmitted sexually from male to female *Ae. albopictus* [62]. Therefore, the increased fecundity of *d5*FWMs may contribute to virus propagation.

This study emphasizes the invasive properties of *Ae. albopictus* and importantly shows the acquisition of an indoor breeding behavior by this major vector of dengue viruses. This behavioral change may lead to increased vectorial capacity. Several parameters come into play when the vectorial capacity of a mosquito for an arbovirus is considered [63]. In particular, host availability and population density are very influential to the competence of a vector. Indeed the more individual vectors are present, the more likely they will be able to transmit a pathogen. Adaption to the indoor/domestic environment, which triggers increased human-vector contacts, will presumably stimulate feeding behavior. In the neighboring Thailand, 100% of field-collected populations of this mosquito fed on humans [64]. Theoretically, such an affinity for feeding on human blood in a wild context will tend to increase inside residences. *Ae. albopictus* exhibited a high biting activity, showing a shorter feeding time and a greater blood feeding success when compared to *Ae. aegypt* at night. This period is the time when residents exhibit low defensive responses to mosquito feeding. In our study, the epidemiological implications were also approached from gonotrophic performance and survival because biting activity is a pivotal factor in the continuation of both pathogen transmission and vector generation [65]. Furthermore, the mosquito individuals that are most easily infected and most likely to incubate a pathogen to an infectious level are those that live long enough [66]. Clearly, adaption to the indoor/domestic environment may produce more competent vectors, since it favors long life and increased lifetime reproductive output.

There is one factor related to our approach that should be discussed in light to keep away from misinterpretations of the obtained results. We have used mice as the blood host. This can appear as a drawback of our method, because the attitudes of a restrained mouse differ from those exhibited by a human under mosquito attacks. We assumed that during sleep, a human may exhibit little defensive responses as a retrained mouse. Experimental mosquitoes were derived from wild pupae collected in a dengue epidemic context. To avoid any infection risks, as no data on dengue infection of *Ae. albopictus* was available, we used mouse as animal model. Although, there are differences in the fitness ramifications for the host species that a mosquito takes blood from, evidence also exist that mice under some conditions mimic well human responses to dengue infection [67], [68].

## References

[1] Burke DS, Monath TP, Knipe DM, Howley PM (2001). Flaviviruses.. Fields Virology. 4th eds.

[2] World Health Organization (2006). Dengue haemorrhagic fever: Early recognition, diagnosis and hospital management – an audiovisual guide for health-care workers responding to outbreaks.. Wkly Epidemiol Rec.

[3] Hawley WA (1988). The biology of *Aedes albopictus*.. J Am Mosq Control Assoc (Suppl.).

[4] Knudsen AB, Romi R, Majori G (1996). Occurrence and spread in Italy of *Aedes albopictus*, with implications for its introduction into other parts of Europe.. J Am Mosq Control Assoc.

[5] Rosen L, Roseboom LE, Gubler DJ, Lien JC, Chaniotis BN (1985). Comparative susceptibility of mosquito species and strains to oral and parenteral infection with dengue and Japanese encephalitis viruses.. Am J Trop Med Hyg.

[6] Mitchell CJ (1995a). Geographic spread of *Aedes albopictus* and potential for involvement in arbovirus cycles in the Mediterranean basin.. J Vector Ecol.

[7] Mitchell CJ (1995b). The role of *Aedes albopictus* as an arbovirus vector.. Parassitologia.

[8] World Health Organization (1999). Prevention and control of dengue and dengue haemorrhagic fever: comprehensive guidelines.. WHO Regional Publication SEARO.

[9] Guzman MG, Kouri G (2002). Dengue: an update.. Lancet Infect Dis.

[10] Deen JL (2004). The challenge of dengue vaccine development and introduction.. Trop Med Int Health.

[11] Guzman MG, Mune M, Kouri G (2004). Dengue vaccine: priorities and progress.. Expert Rev Anti Infect Ther.

[12] Pang T (2003). Vaccines for the prevention of neglected diseases-dengue fever.. Curr Opin Biotechnol.

[13] Fay RW, Eliason DA (1966). A preferred oviposition site as a surveillance method for *Aedes aegypti*.. Mosq News.

[14] Ritchie SA, Pyke AT, Smith GA, Northhill JA, Hall RA (2003). Field evaluation of a sentinel mosquito (Diptera: Culicidae) trap system to detect Japanese Encephalitis in remote Australia.. J Med Entomol.

[15] Reiter P (1986). A standardized procedure for the quantitative surveillance of certain *Culex* mosquitoes by egg raft collection.. J Am Mosq Control Assoc.

[16] Savage HM, Anderson M, Gordon E, McMillen L, Colton L (2008). Host-seeking heights, host-seeking activity patterns, and West Nile virus infection rates for members of the *Culex pipiens* complex at different habitat types within the hybrid zone, Shelby County, TN, 2002 (Diptera : Culicidae).. J Med Entomol.

[17] Roitberg BD, Sircom J, Roitberg CA, Vanalphen JJM, Mangel M (1993). Life expectancy and reproduction.. Nature (Lond.).

[18] Leisnham PT, Sala LM, Juliano SA (2008). Geographic variation in adult survival and reproductive tactics of the mosquito *Aedes albopictus*.. J Med Entomol.

[19] Chow VTK, Chan YC, Yong R, Lee KM, Lim LK (1998). Monitoring of dengue viruses in field-caught *Aedes aegypti* and *Aedes albopictus* mosquitoes by a type-specific Polymerase Chain Reaction and cycle sequencing.. Am J Trop Med Hyg.

[20] Ali M, Wagatsuma Y, Emch M, Breiman RF (2003). Use of a geographic information system for defining spatial risk for dengue transmission in Bangladesh: role for *Aedes albopictus* in an urban outbreak.. Am J Trop Med Hyg.

[21] Almeida APG, Baptista SSGS, Sousa AGCC, Novo MTLM, Ramos HC (2005). Bioecology and vectorial capacity of *Aedes albopictus* (Diptera: Culicidae) in Macao, China, in relation to dengue virus transmission.. J Med Entomol.

[22] Rudnick A, Tan EE, Lucas JK, Omar MB (1965). Mosquito borne haemorrhagic fever in Malaysia.. Brit Med J.

[23] Nur Aida H, Abu Hassan A, Nurita AT, Che Salmah MR, Norasmah B (2008). Population analysis of *Aedes albopictus* (Skuse) (Diptera:Culicidae) under uncontrolled laboratory conditions.. Trop Biomed.

[24] Rozilawati H, Zairi J, Adanan CR (2007). Seasonal abundance of *Aedes albopictus* in selected urban and suburban areas in Penang, Malaysia.. Trop Biomed.

[25] Ahmad F, Ahmad SY, Farooqi MA (2006). Characterization and geotechnical properties of Penang residual soils with emphasis on landslides.. Am J Environ Sciences.

[26] Thavara U, Tawatsin A, Chansang C, Kong-ngamsuk W, Paosriwong S (2001). Larval occurrence, oviposition behavior and biting activity of potential mosquito vectors of dengue on Samui Island, Thailand.. J Vector Ecol.

[27] Chadee DD, Martinez R (2000). Landing periodicity of *Aedes aegypti* with implications for dengue transmission in Trinidad, West Indies.. J Vector Ecol.

[28] Kawada H, Takemura SY, Arikawa K, Takagi M (2005). Comparative Study on Nocturnal Behavior of *Aedes aegypti* and *Aedes albopictus*.. J Med Entomol.

[29] Tanaka K, Mizusawa K, Saugstad ES (1979). A revision of the adult and larval mosquitoes of Japan (including the Ryukyu Archipelago and the Ogasawara Islands) and Korea (Diptera, Culicidae).. Contribut Am Entomol Inst.

[30] Goma LKH (1966). The Mosquitoes.

[31] Blackmore MS, Lord CC (2000). The relationship between size and fecundity in *Aedes albopictus*.. J Vector Ecol.

[32] Chadee DD, Beier JC, Mohammed RT (2002). Fast and slow blood-feeding durations of *Aedes aegypti* mosquitoes in Trinidad.. J Vector Ecol.

[33] Systat® 11 software (2004). Systat 11 for windows: Statistics.

[34] Makiya K (1968). Population dynamics of larvae overwintering in southern Japan.. Japn J Sanit Zool.

[35] Sota T, Mogi M, Hayamizu E (1992). Seasonal distribution and habitat selection by *Aedes albopictus* and *Aedes riversi* (Diptera : Culicidae) in Northern Kyushu, Japan.. J Med Entomol.

[36] Preechaporn W, Mullica J, Jaroensutasinee K (2006). The larval ecology of *Aedes aegypti* and *Ae. albopictus* in three topographical areas of Southern Thailand.. Dengue Bull.

[37] Dye C (1992). The analysis of parasite transmission by blood-sucking insects.. Annu Rev Entomol.

[38] McCrae AW (1983). Oviposition by African malaria vector mosquitoes. I. Temporal activity patterns of caged, wild-caught, freshwater *Anopheles gambiae* Giles sensu lato.. Ann Trop Med Parasitol.

[39] Chadee DD, Corbet PS (1990). A night-time role of the oviposition site of the mosquito, *Aedes aegypti* (L) (Diptera: Culicidae).. Ann Trop Med Parasitol.

[40] Clements AN (1999). The biology of mosquitoes, volume 2: Sensory reception and behaviour.

[41] Sumba LA, Okoth K, Deng AL, Githure J, Knols BGJ (2004). Daily oviposition patterns of the African malaria mosquito *Anopheles gambiae* Giles (Diptera: Culicidae) on different types of aqueous substrates.. J Circadian Rhythms.

[42] Grech K, Maung LA, Read AF (2007). The effect of parental rearing conditions on offspring life-history in *Anopheles stephensi*.. Malar J.

[43] Nayar JK, Sauerman DM (1975). Physiological basis of host susceptibility of Florida mosquitoes to *Dirofilaria immitis*.. J Insect Physiol.

[44] Chan KL, Ho BC, Chan YC (1971). *Aedes aegypti* (L) and *Aedes albopictus* (Skuse) in Singapore City. 2. Larval habitats.. Bull WHO.

[45] Waterman SH, Novak RJ, Sather GE, Bailey RE, Rios L (1985). Dengue transmission in two Puerto Rican communities in 1982.. Am J Trop Med Hyg.

[46] Goh KT, Ng SK, Chan YC, Lim SJ, Chua EC (1987). Epidemiological aspects of an outbreak of dengue fever/dengue haemorrhagic fever in Singapore.. Southeast Asian J Trop Med Pub Health.

[47] Focks DA, Brenner RJ, Hayes J, Daniels E (2000). Transmission thresholds for dengue in terms of *Aedes aegypti* pupae per person with discussion of their utility in source reduction efforts.. Am J Trop Med Hyg.

[48] Rodriguez-Figueroa L, Rigau-Perez JG, Suarez EL, Reiter P (1995). Risk factors for dengue infection during an outbreak in Yanes Puerto Rico in 1991.. Am J Trop Med Hyg.

[49] Dengue (1992). Seroprevalence of dengue virus infection. Singapore.. Wkly Epidemiol Rec.

[50] Pontes RJ, Freeman J, Oliveira-Lima JW, Hodgson JC, Spielman A (2000). Vector densities that potentiate dengue outbreaks in a Brazilian city.. Am J Trop Med Hyg.

[51] Moore CG, Cline BL, Ruiz-Tiben E, Lee D, Romney-Joseph H (1978). *Aedes aegypti* in Puerto Rico: environmental determinants of larval abundance and relation to dengue virus transmission.. Am J Trop Med Hyg.

[52] Teixeira Mda G, Barreto ML, Costa Mda C, Ferreira LD, Vasconcelos PF (2002). Dynamics of dengue virus circulation: a silent epidemic in a complex urban area.. Trop Med Int Health.

[53] Effler PV, Pang L, Kitsutani P, Vorndam V, Nakata M (2005). Dengue fever, Hawaii, 2001–2002.. Emerg Infect Dis.

[54] Woke PA (1937). Effects of various blood fractions on egg production of *Aedes aegypti* Linn.. Am J Hyg.

[55] Turley AP, Moreira LA, O'Neill SL, McGraw EA (2009). *Wolbachia* infection reduces blood-feeding success in the dengue fever mosquito, *Aedes aegypti*.. PLoS Negl Trop Dis.

[56] Briegel H (1990). Fecundity, metabolism, and body size in *Anopheles* (Diptera: Culicidae) vectors of Malaria.. J Med Entomol.

[57] Klowden MJ, Lea OA (1978). Blood meal size as a factor affecting continued host-seeking by *Aedes aegypti* (L.).. Am J Trop Med Hyg.

[58] Clements AN (1992). The biology of mosquitoes. Volume 1: development, nutrition and reproduction.

[59] Foster WF (1995). Mosquito sugar feeding and reproductive energetics.. Annu Rev Entomol.

[60] Reisen WK, Takken W, Scott TW (2003). Lessons from the past: historical studies by the University of Maryland and the University of California, Berkeley.. Ecological aspects for application of genetically modified mosquitoes.

[61] Briegel H, Timmermann SE (2001). *Aedes albopictus*: physiological aspects of development and reproduction.. J Med Entomol.

[62] Rosen L (1987). Sexual transmission of dengue viruses by *Aedes albopictus*.. Am J Trop Med Hyg.

[63] Reisen WK (1989). Estimation of vectorial capacity: relationship to disease transmission by malaria and arbovirus vectors.. Bull Soc Vector Ecol.

[64] Ponlawat A, Harrington LC (2005). Blood feeding patterns of *Aedes aegypti* and *Aedes albopictus* in Thailand.. J Med Entomol.

[65] Hurd H, Warr E, Polwart A (2001). A parasite that increases host lifespan.. Proc Roy Soc Lond Series B- Biol Sciences.

[66] Sumanochitrapon W, Daniel S, Sithiprasana R, Kittayapong P, Innis BL (1998). Effect of size and geographic origin of *Aedes aegypti* on oral infection with dengue virus-2.. Am J Trop Med Hyg.

[67] Lin Y-L, Liao C-L, Chen L-K, Yeh CT, Liu CI (1998). Study of dengue virus infection in SCID mice engrafted with human K562 Cells.. J Virol.

[68] Huang K-J, Li S-YJ, Chen S-C, Liu H-S, Lin Y-S (2000). Manifestation of thrombocytopenia in dengue-2-virus-infected mice.. J Gener Virology.

